# Enhanced Passive RF-DC Converter Circuit Efficiency for Low RF Energy Harvesting

**DOI:** 10.3390/s17030546

**Published:** 2017-03-09

**Authors:** Issam Chaour, Ahmed Fakhfakh, Olfa Kanoun

**Affiliations:** 1 Chair of Measurement and Sensor Technology, Technische Universität Chemnitz, 09126 Chemnitz, Germany; olfa.Kanoun@etit.tu-chemnitz.de; 2 National Engineering School of Sfax, University of Sfax, Sfax 3038, Tunisia; 3 National School of Electronic and Telecommunication (ENET’Com), Sfax 3018, Tunisia; ahmed.fakhfakh@isecs.rnu.tn

**Keywords:** radio frequency energy transmission, RF-DC Converter, rectifier, power efficiency, voltage multiplier, Villard topology, Dickson topology

## Abstract

For radio frequency energy transmission, the conversion efficiency of the receiver is decisive not only for reducing sending power, but also for enabling energy transmission over long and variable distances. In this contribution, we present a passive RF-DC converter for energy harvesting at ultra-low input power at 868 MHz. The novel converter consists of a reactive matching circuit and a combined voltage multiplier and rectifier. The stored energy in the input inductor and capacitance, during the negative wave, is conveyed to the output capacitance during the positive one. Although Dickson and Villard topologies have principally comparable efficiency for multi-stage voltage multipliers, the Dickson topology reaches a better efficiency within the novel ultra-low input power converter concept. At the output stage, a low-pass filter is introduced to reduce ripple at high frequencies in order to realize a stable DC signal. The proposed rectifier enables harvesting energy at even a low input power from −40 dBm for a resistive load of 50 kΩ. It realizes a significant improvement in comparison with state of the art solutions.

## 1. Introduction

The use of energy harvesting technologies in wireless sensor networks (WSN) enables reducing installation and maintenance efforts for autonomous systems [1]. It helps to overcome many challenges, such as difficult direct access, flexible use, and working under harsh environmental conditions. Nevertheless, in some cases, using only energy harvesting [2] is not sufficient because of the lack of availability of ambient energy, aging effects, and changing ambient conditions. 

Wireless power transmission provides a potential solution to support energy harvesting as an additional source and is able to bridge a relatively long distance between a sensor node and its power source [3]. The transmitted radio frequency (RF) power can reach a level of microwatts (μW) to milliwatts (mW) DC, which is a useful range for charging batteries or powering battery-free devices.

The main challenge of RF energy transmission is the low level of the received power and the RF-DC power conversion efficiency (PCE). In this paper, we investigate possibilities to improve the efficiency at the receiver side. In order to be able to convert RF energy even from low power ambient RF sources, it is necessary to optimize the system design, especially for low RF energy density. Therefore, we focus on the rectifier circuit, due to the interesting possibilities to reduce energy losses. 

This paper is structured in three main sections. In Section 2, a short description of the related challenges and solutions for RF power transmission is presented followed by a discussion on RF energy conversion and storage. Section 3 describes the Villard voltage rectifier circuit for RF-DC converters and discusses the component selection. Finally, the novel approach for an RF-DC voltage multiplier rectifier circuit is detailed, illustrating the simulation results and undertaking the proved circuit performance.

## 2. RF Energy Conversion and Storage 

**Figure 1:**
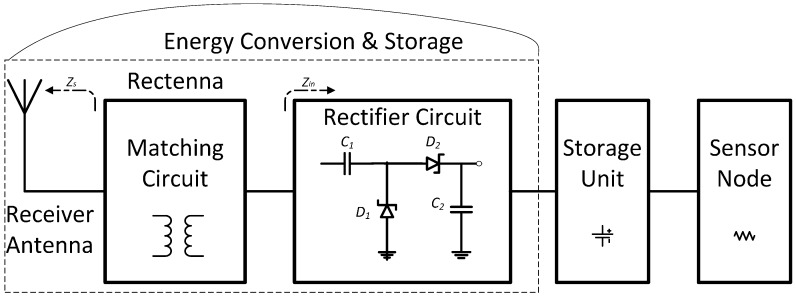
RF energy harvesting sensor design.

RF transfer requires sophisticated circuits for conversion and storage the available RF ambient energy on the receiver side [4]. As shown in Figure 1, this can be reached by the optimization interface between the rectenna (rectifying antenna), and typical storage unit for the WSN. The main aim is to reach a high overall efficiency by minimizing discontinuities and signal reflections [5]. For that, a reactive matching circuit connects the antenna to the rectifier under optimized operating conditions [6]. 

RF wave propagation models are available to evaluate the received RF power. The received power (*P_r_*) by the antenna placed at a distance *R* from the transmitter antenna can be expressed as in Equation (1) [7].

(1) $$P_{r}=P_{t}G_{t}G_{r}{\left(\frac{C}{4\pi f}\right)}^{2}{\left(\frac{1}{R}\right)}^{n}e^{-\alpha R}$$

where *P_t_* is the power transmitted which corresponds to the input power to the transmitter antenna, *G_t_* and *G_r_* are, respectively, the gains of receiving and transmitting antennas, *C* is the speed of light, and *f* is the RF signal frequency. *α* is the effective decay coefficient in air and is equal to 0.001. *n* denotes the path loss exponent and is equal to 2 in free space. As it is shown *P_r_* depends on multiple factors: the power of RF source, size/performance of receiving antenna, transmission frequency and the distance from the RF source [8].

Since special regulations exist for RF power transmission, it makes sense to use the free license frequency bands, or industrial, scientific, and medical bands (ISM). They should be classified as either nonspecific short-range devices (SRD), wideband data transmission systems, or radio frequency identification (RFID) applications [9,10]. For example, 867.6–868 MHz band is one of the RFID frequency ranges used in ultra-high frequency SRD applications. For this band, it is allowed to transfer until 500 mW effective radiated power (ERP) in Europe with 200 kHz of coupling channel spacing [11]. 

Many rule parts specify RF power transmission limits in terms of ERP or equivalent isotropically radiated power (EIRP). ERP and EIRP are defined in linear terms as the product of the antenna gain and the input power. 

A lot of calculations and analyses are required to investigate the efficiency and performance of RF power transfer. After this approximate study, it is basic to note that increasing frequency leads to a reduction in the received power level, hence, the decrease in the yield. To solve this problem, studying the rectification systems characterized by low power loss and high efficiency to recover the maximum RF energy transmitted is needed.

## 3. Basic Rectifier Circuit for RF Applications

An impedance matching circuit between the received aerial and rectifier circuit is necessary to increase the voltage gain and further reduce reflection and transmission loss. For low-power and sensing applications, the main aim to satisfy after rectification is to recover the maximum amount of power and reduce the power loss caused by the rectifier circuit. Many investigations showed that when the applied power is low, the rectifier circuit efficiency is also low. Therefore, in order to rectify low RF signals at a high efficiency, it is interesting to improve the rectifier [12]. Power-conversion efficiency (PCE) or RF-to-DC conversion efficiency, is an important rectification metric for optimal wireless power transmission [13] and is calculated as follow Equation (2):

(2) $$\mathrm{PCE}=\frac{\mathrm{DC}\;\mathrm{Output}\;\mathrm{Power}}{\mathrm{Incident}\;\mathrm{RF}\;\mathrm{Power}-\mathrm{Reflected}\;\mathrm{RF}\;\mathrm{Power}}$$

The voltage multiplier structure is considered for RF-DC power conversion system design because it rectifies peak-to-peak voltage from the full-wave of the RF signal. Two configurations are arranged in a cascade using Schottky diodes to provide a passive voltage offset before rectification [14]. The conventional voltage multiplier rectifier forms a peak rectified by *D*_1_ and *C*_2_, while a voltage clamp is formed by *C*_1_ and *D*_2_.

The circuit can be also called a voltage doubler, thereby, the output voltage is approximately twice the input voltage. The RF input signal is rectified during the positive alternative. The stored voltage on the input capacitor *C*_1_ during the negative alternative is transmitted to the output capacitor *C_2_* during the next positive alternative of the RF input signal. Thus, the voltage on *C*_2_ is roughly two times the peak voltage of the RF source minus two times the turn on voltage of the diode [15]. 

**Figure 2:**
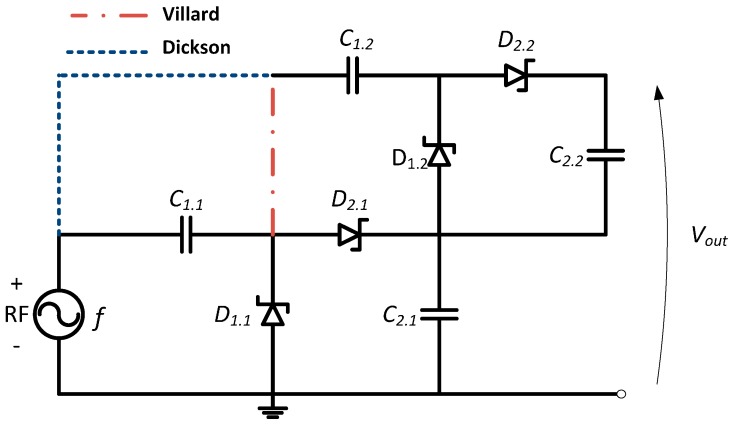
Two-stage Villard and Dickson configuration for a combined voltage multiplier rectifier circuit.

One voltage doubler circuit can be extended to n stages in cascade to achieve higher DC output voltage levels. In the Villard topology the stages are connected in series and behave similarly to batteries in cascade, multiplying the output voltage. Figure 2 illustrates a two stages Villard voltage multiplier circuit. Each Villard stage acts as a passive voltage shifter producing a DC offset voltage for the next stage. 

The stage number used in the rectifier has a major influence on the harvesting circuit. Since one rectifier stage may yield to an unused output voltage and too many Villard stages dampens the multiplier effect by reducing the impedance reactive component. Thus, practical constraints oblige a limitation on the permitted stages due to the capacitor parasitic effect of each stage. Thus, the voltage can be increased by increasing the circuit stage number, which increases the power loss.

The circuit is efficient only when the coupling capacitances (*C*_1.1_, *C*_1.2_…*C*_1*.n*_) are higher than the connection capacity to each circuit node (*C*_2.1_, *C*_2.2_…*C*_2*.n*_). In addition, the capacitors present a high parasitic capacitance relative to the substrate. To overcome these problems, Dickson proposed the circuit presented in Figure 2 [16]. It is characterized by an effective voltage multiplication with relative low parasitic effects [17]. A comparison between the two architecture topologies was performed in [18]. For low voltages, the two circuits offer similar performance.

## 4. Novel Approach for an RF-DC Converter Based on Voltage Multiplier Circuit

**Figure 3:**
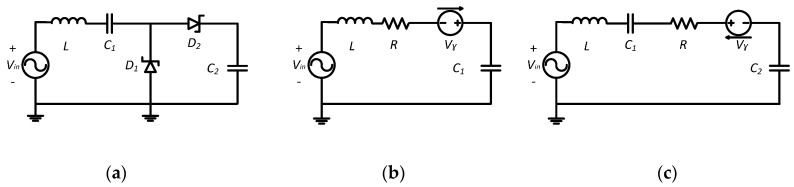
(**a**) Novel approach for a voltage multiplier circuit; (**b**) Equivalent circuit during the positive wave; (**c**) Equivalent circuit during the negative wave.

The principal aim is energy harvesting from low RF density waves available at 868 MHz. The received RF power generally ranges from 0 to −30 dBm relative to transmitted power. We propose adding an inductor *L* in series with *C*_1_ (Figure 3) in order to store the energy in a magnetic field. Energy is stored during the negative cycle wave and returned during the positive one. The couple (*L*, *C*_1_) operates like an additional power source during the positive alternation [19]. The power stored in the inductance magnetic field is given by the following relation, Equation (3), in terms of the current *i*:

(3) $$P=L\;i\;\frac{di}{dt}$$

### 4.1. Novel Approach for a Signal Voltage Multiplier Circuit

**Figure 4:**
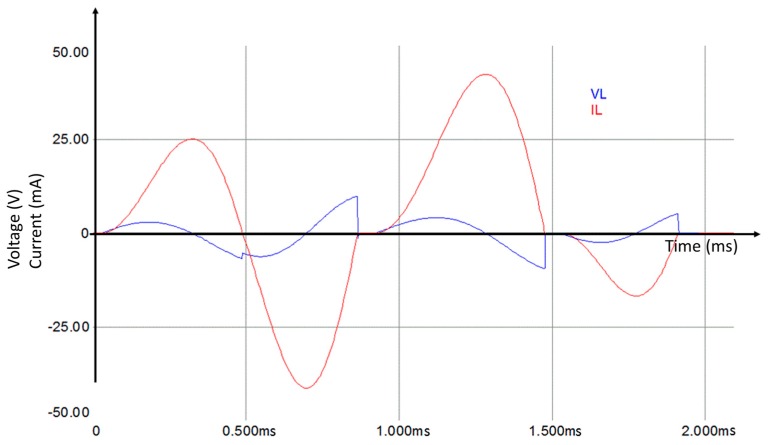
Current and voltage of the added inductor.

To explain the principle of this approach, we have carried out a simulation of the circuit at low frequency with standard elements using Simplorer software. Figure 4 illustrates the inductor voltage and current during the transitional period. 

**Figure 5:**
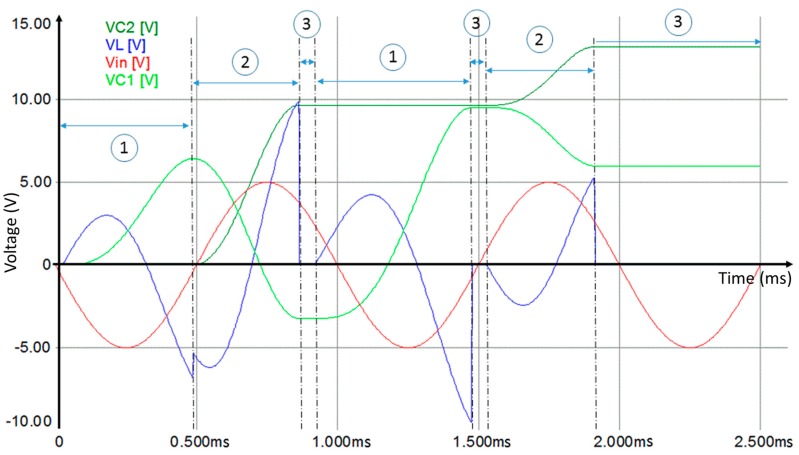
Voltage behavior of the rectifier circuit elements.

The current passing through the inductor is always continuous. The potential of the inductor is not proportional to the inductor current. The current passing through the inductor is constantly charging and discharging. Therefore, the inductor generates an opposite voltage polarity in order to limit the current variation [20]. This potential can be used to charge the capacitances *C*_1_ and *C*_2_. Current and voltage discontinuity, presented in Figure 4, is caused by the status variation of diodes *D*_1_ and *D*_2_. The simulation results show the voltage behavior of the circuit elements for different time slots (Figure 5).

During phase 1, *D*_2_ is blocked until the condition given by Equation (4) is fulfilled:

(4) $$V_{C1}>-V_{in}-V_{l}-V_{\gamma }-Ri$$

*C*_1_ and *C*_2_ are equal to 1 mF. The negative alternation of the input voltage (*V_in_* equal 5 V, the frequency is equal to 1 kHz) crosses *D*_1_ to charge the capacitor *C*_1_ and the inductor *L*. We denote that *R* is equal to 0.1 Ω, which is the internal resistance, and *V_ɣ_* is equal to 0.8 V, which is the threshold voltage of the diodes. The variation of the inductor current *i* causes an opposite voltage polarity *V_L_* across its terminals. The voltage *V_C_*_1_ across *C*_1_ is determined by Equation (5):

(5) $$V_{C1}=-V_{in}-V_{l}-V_{\gamma }-Ri$$

During phase 2, *D*_1_ is blocked and the capacitance *C*_1_ discharges through *D*_2_ to charge the capacitor *C*_2_. The voltage across C_2_ is determined by Equation (6):

(6) $$V_{C2}=V_{C1}+V_{in}+V_{l}-V_{\gamma }-Ri$$

The system change the status from phase 2 to another phase when Equation (7) is fulfilled:

(7) $$V_{C2}>V_{C1}+V_{in}+V_{l}-V_{\gamma }-Ri$$

During phase 3, both diodes *D*_1_ and *D*_2_ are blocked as long as Equations (4) and (7) are verified. The charging cycle of *C*_1_ or *C*_2_ restarts again depending on the conditions established by Equations (4) or (7). At the end, the system is stable. *C*_1_ and *C*_2_ maintain the charge level.

**Figure 6:**
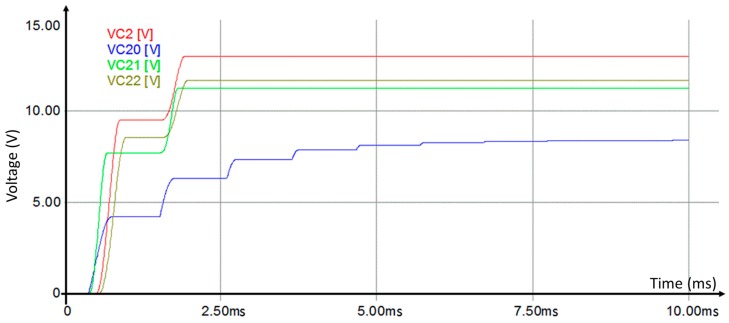
Comparison between the classic voltage multiplier (*V_C_*_20_) and the novel approach with different inductor values (*L*, *L_1_* = *L* + Δ*L*, and *L_2_* = *L* − Δ*L* corresponding to *V_C_*_2_, *V_C_*_21_, and *V_C_*_22_).

The proposed approach aims to ameliorate the charging of capacitances *C*_1_ and *C*_2_ using the opposite voltage polarity generated by the inductor *L*. Figure 6 shows a comparison between the classic voltage multiplier circuit and the novel approach. The novel approach provides higher output voltage (*V_C_*_2_) and exceeds the classic voltage multiplier circuit (*V_C_*_20_). The system establishing time is reduced by the same inductor. Simulations with varied inductor values show a better performance for L equal to 0.023 mH (Figure 6). 

The first sub-circuit of Figure 3 represents a second order system. The solution of its equations leads to determine the charging expression of *C*_1_, as defined in Equation (8):

(8) $$\begin{array}{l} V_{C1}(t)=e^{-\lambda t}\left[(V\gamma -V\sin\psi \text{​})\cos\omega t+\frac{\lambda (V\gamma -V\sin\psi \text{​})-V\omega_{in}\cos\psi }{\omega }\sin\omega t\right]+\\ V\sin(\omega_{in}t+\psi )-V_{\gamma } \end{array}$$

Where *V* (Equation (9)) and *ѱ* (Equation (10)) are, respectively, the amplitude and the phase of the particular solution.

(9) $$V=\frac{V_{in}}{\sqrt{{C_{1}}^{2}{\omega_{in}}^{2}R^{2}+{\left(C_{1}L{\omega_{in}}^{2}-1\right)}^{2}}}$$

(10) $$\psi =\pi -arcsin\left(\frac{RC_{1}\omega_{in}}{\sqrt{{C_{1}}^{2}{\omega_{in}}^{2}R^{2}+{\left(C_{1}L{\omega_{in}}^{2}-1\right)}^{2}}}\right)$$

The damping coefficient *λ* is defined in Equation (11):

(11) $$\lambda =\frac{R}{2L}$$

The comparison between the simulation and the analytical expression validates the expression of *V_C_*_1_ during phase 1. For phase 2, *C*_1_ is discharging and *C*_2_ is charging. From the simulation results, *V_C_*_1_ and *V_C_*_2_ are characterized with the same slope but in opposite sign (negative for *V_C_*_1_ and positive *V_C_*_2_). In order to determine the expression of the optimal inductor value from the expression of *V_C_*_2_ max, it is necessary to express the initial charge of *C*_1_ for phase 2. Since it is difficult to solve such equations with multiple transcendental functions, we propose to solve it with numerical methods. After multiple simulations, with a varied input parameters, the result show that for *ɷ* equal *ɷ_in_* the system has a higher outcome. Where *ɷ_in_* is the pulse of the input signal and *ɷ* is defined in Equation (12):

(12) $$\omega =\sqrt{\frac{1}{LC_{1}}-\frac{R^{2}}{4L^{2}}}$$

Based on these simulations results, it is easy to give an optimal expression estimation of *L* (Equation (13)):

(13) $$L=\frac{1+\sqrt{1-C^{2}R^{2}{\omega_{in}}^{2}}}{2C{\omega_{in}}^{2}}$$

### 4.2. Proposed Signal Voltage Multiplier Circuit

**Figure 7:**
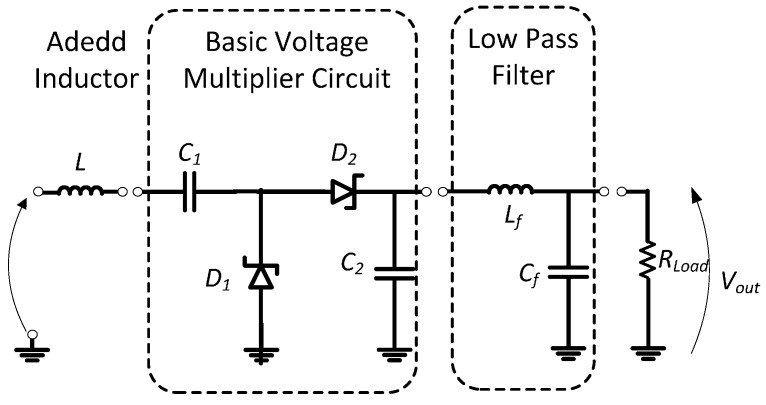
Proposed single stage voltage multiplier RF-DC converter.

The incident RF signal waves captured by the receiver antenna are converted into a DC signal by a simple topology based on a voltage multiplier used for the RF-DC converter circuit. The major feature of this architecture is just to multiply the input voltage to its output terminal using the continuous voltage component of the previous stage adding to the rectified RF signal of the next stage. The multiplier circuit is designed using two zero-based HSMS-2850 Schottky diodes characterized by low threshold voltage and high switching operating frequency [21]. The output voltage depends on the storage capacitance. The capacitors are chosen with a lower value to reduce the charging time and rise of the switching speed of the whole circuit. Figure 7 presents one stage circuit design tuned to the unlicensed ISM band at 868 MHz. In order to obtain good DC output and reduce ripples, a low pass filter is associated in cascade to the RF-DC converter circuit. The output inductor and capacitor are chosen to filter the high frequencies. 

**Figure 8:**
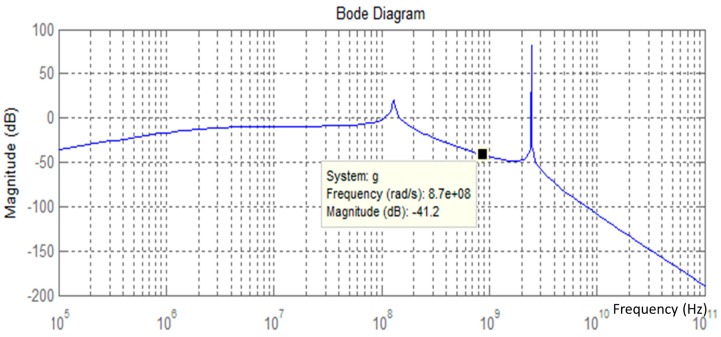
Frequency response behavior of the proposed rectifier design.

A 50 kΩ load is connected at the output to measure the outcome power and to visualize the circuit behavior (Figure 8). The load value is chosen relative to the sensor node impedance in a deep sleep state. For the operating frequency, the signal is well filtered with an attenuation of −40 dB.

### 4.3. Simulations for a Modified Voltage Multiplier Circuit

**Figure 9:**
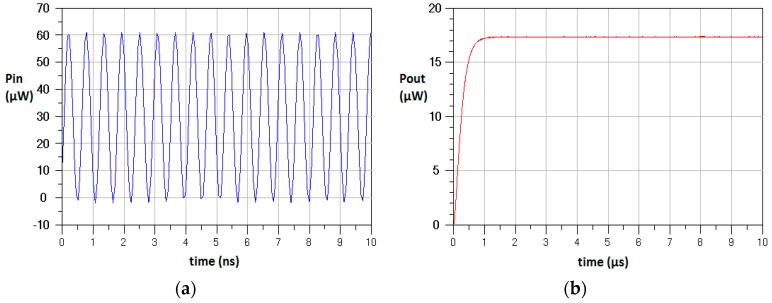
Simulation results of the proposed rectifier design. (**a**) Input RF wave form; (**b**) Output power after the rectifier circuit.

The modified voltage doubler circuit is simulated using Advanced Design System (ADS) from Agilent. The ADS simulation employs the device model of HSMS-2850 Schottky diode parameters and achieved the results presented in Figure 9.

**Figure 10:**
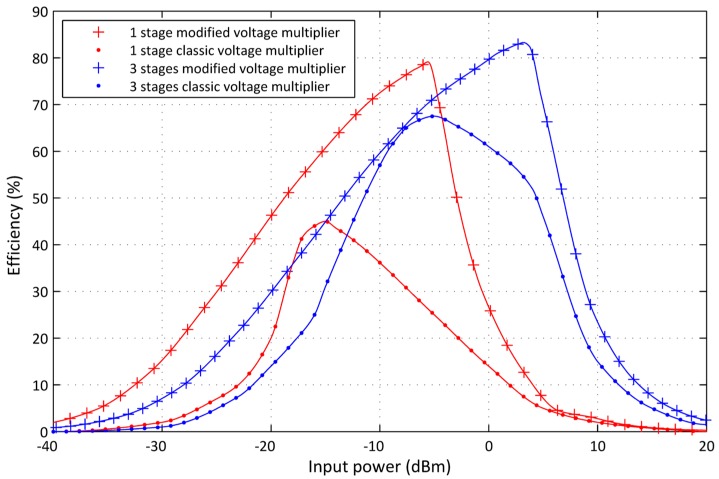
Comparison between the proposed rectifier design efficiency and the classic voltage multiplier efficiency.

To discuss the RF-DC converter circuit performances, simulations were carried out. The results presented in Figure 10 show the conversion efficiency for one and three multiplier stages for variable input power from −40 to 20 dBm. The proposed design with inductor is compared to the classic voltage multiplier circuit without inductor for a resistive load of 50 kΩ. The novel design reaches an efficiency which is much higher than the classic voltage multiplier circuit, especially for low input power. With one stage, the novel design reaches greater efficiency, with a peak of 79%. With three stages, the novel design reaches 83% peak efficiency. The novel RF-DC converter circuit consists of diodes, which have nonlinear component behavior. In addition, the circuit design itself exhibits nonlinear effects due to the parasitic influence of the used elements. This implies that the response of the harvester circuit varies with the received power amount delivered by the antenna. RF input power variation does not correlate with the output power of the harvesting circuit. 

**Figure 11:**
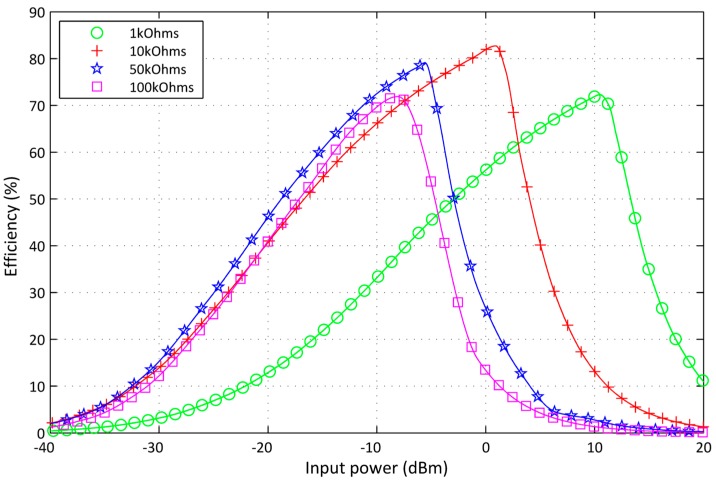
Load influence on the signal stage modified voltage multiplier efficiency.

The output load of the RF-DC converter circuit needs to be improved using harmonic balance in order to enhance the PCE. The output DC power is measured around 50 kΩ resistive load. Figure 11 illustrates the load influence on one stage RF-DC converter circuit PCE. The previous simulation parameters and a variable resistive load are used to investigate the load impact on the PCE. Referred to the sensor node impedance and according to sleep mode, the proposed RF-DC converter is loaded by 50 kΩ.

**Figure 12:**
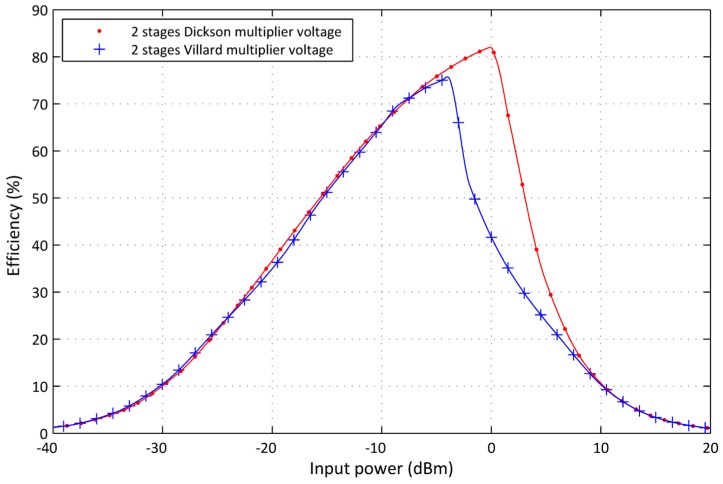
Comparison between Dickson and Villard topologies.

The novel designed RF-DC converter is capable to convert an RF wave to a DC signal and achieving higher efficiency based on passive elements. Simulations are conducted in ADS under the same scenarios in order to prove one voltage multiplier topology from Villard or Dickson configurations. Figure 12 shows the simulation results for two stages Dickson configuration compared to two stages Villard configuration. The Dickson topology efficiency surpasses Villard topology efficiency all over the full input power range. The Dickson architecture is selected as a parallel capacitor configuration to reduce losses in each stage.

**Figure 13:**
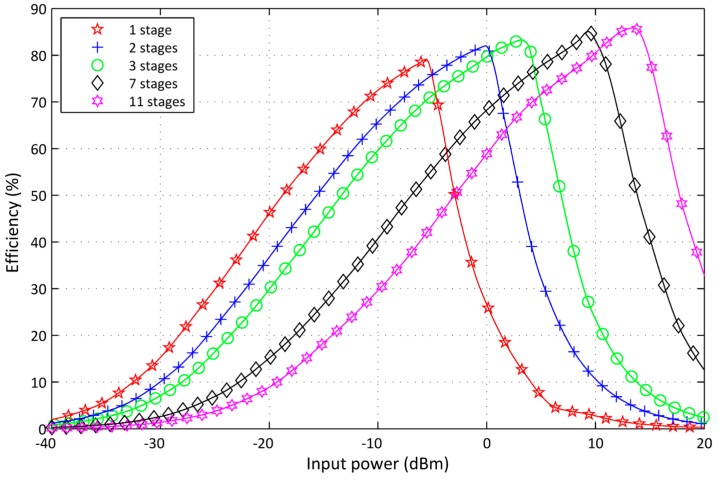
Effect of the stage number on the power conversion.

The number of RF-DC converter stages has a signficant effect on the circuit output power. The voltage multiplier stages are reformed and arranged in cascade. The output power is directly proportional to the stage number and the input power. However, practical constraints limit the approved stages number and, therefore, the output power. Sweep input power parameters from −40 to 20 dBm was used in simulations with a variety of stage numbers from 1 to 11 stages. Figure 13 shows the impact of the stages number on PCE and the output power of the new RF-DC converter circuit design. Finally, the PCE variation becomes negligible. However, when the stage number increases, the efficiency curve shifts towards the higher input power region. This means an increase of the power losses in the region of low input power.

### 4.4. Design and Experiment Setup for a Modified Dual Stages Voltage Multiplier Circuit

**Figure 14:**
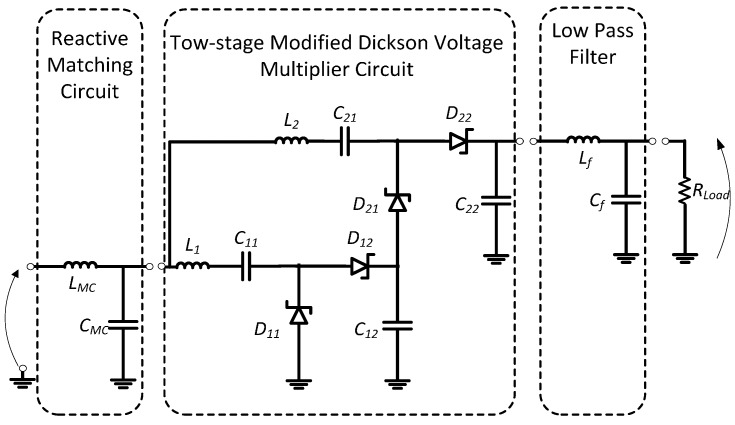
Dual-stage proposed RF–DC converter circuit.

The receiver antenna is connected to the RF-DC converter block via a reactive matching circuit in order to efficiently load and increase the voltage gain. This allows a decrease in the reflected signal and, therefore, losses. Figure 14 presents a two-stage voltage multiplier circuit. The return loss S_11_ parameter and the circuit impedance are measured relative to the input RF power at −10 dBm and the effective frequency matched to 50 Ω. 

**Figure 15:**
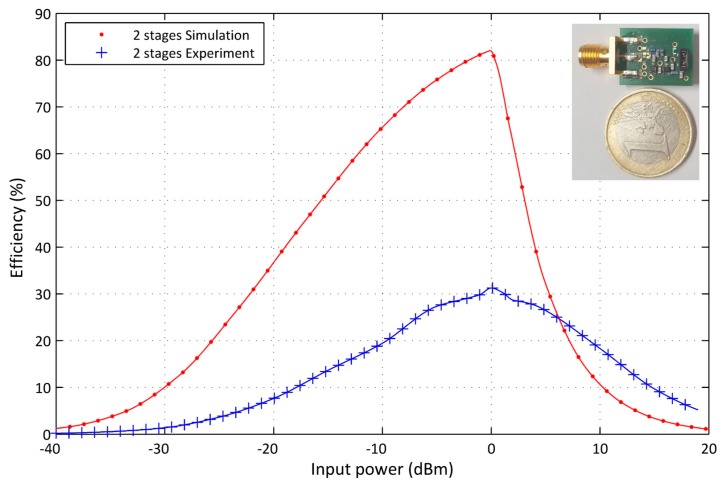
Comparison between simulation and experimental PCE results of the proposed RF–DC converter.

Experiment results prove that the circuit design runs at 868 MHz. The final modified RF-DC converter printed circuit board (PCB), shown in Figure 15, is fabricated using FR-4 substrate. It contains two layers, one electrically connects the passive components and the second is a ground plane. A large deviation between simulation and experiment results is shown in Figure 15. This divergence is caused by components’ tolerances and the parasitic capacitance produced by the circuit layout. PCB layers and non-ideal component behaviour generate upper losses, particularly, for high frequency and the low input power range. The HSMS-2850 library model is being supplied for simulation to reflect typical baseline aspects. Certain performance modulations miss the perfection compared to the real Schottky diode behavior. The model limitation and the sensitivity of Schottky diode parameters to the ambient temperature produce a mismatch between the simulation and experimental results. 

At −10 dBm input power the measured PCE of the modified RF-DC converter is about 19.49%. The proposed design provides 19.43 µW output power and around 1 V output voltage. The circuit achieves 26.21% of PCE at −6 dBm. It delivers 78.72 µW and reaches 2 V output voltage. This means the proposed modified RF-DC converter design can power a diversity of microcontrollers. Among them, the MSP430L092, which runs with 0.9 V with 6 µW power consumption in standby mode and 3 µW in off mode. It consumes 45 µA in active mode and works at 1.3 V [22].

**Figure 16:**
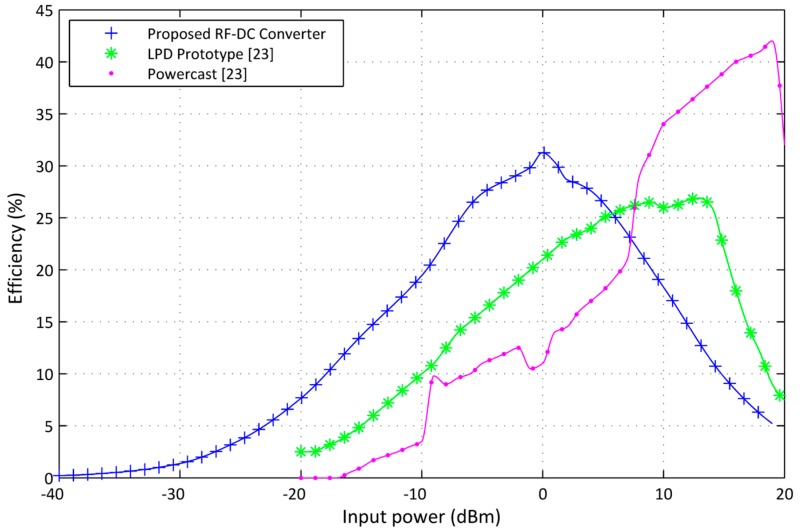
Comparison between PCE results of the proposed RF-DC converter, LPD prototype from [23], and the commercial RF energy harvester Powercast P1100 [23].

In order to evaluate the efficiency of the proposed dual-stage design, we compare archived experimental results to the state of the art presented in [23]. LPD, presented in [23], is a low power design harvester consisting of a seven-stage classic voltage multiplier circuit connected to a 100 kΩ load. It operates at 915 MHz and employs Agilent HSMS-2852 Schottky diodes, which are used for our proposed design. The setup results of the LDP, as published in [23], are presented in Figure 16. LPD-PCE is about 10% at −10 dBm input power and around 15% at −6 dBm. Consequently, this performance evaluation purpose shows a comparison of LPD-PCE and the proposed dual-stage design. The proposed dual-stage design provides higher efficiency. The performance of the proposed dual-stage design stands out in the low power region where the LPD efficiency is low. The main target of this RF energy harvesting system is to yield energy of a low RF density area. The incident RF power in the energy harvesting case is very low. The input power range is limited and rarely exceeds 0 dBm for ambient RF energy harvesting. In [23] a comparison of LPD with the commercial RF energy harvester from a Powercast P1100 is carried out. Figure 16 shows a PCE of P1100 across the 100 kΩ load [23]. The comparison demonstrates that the proposed design leads to a higher PCE than the P1100 at low input power.

## 5. Conclusions

With energy harvesting and transfer, it is possible to recover micro-power, useful for powering low-power wireless sensor nodes. In this work, a passive ultra-low RF-DC converter circuit design operating at 868 MHz is presented. It is capable of harvesting low ambient RF power levels using a novel multiplier circuit technique and high quality components to reduce parasitic effects and threshold voltages. New techniques are used for the modified voltage multiplier circuit. They consist of including an inductor in series with the input capacitance, which is able to enhance the PCE of the design. 

The proposed RF-DC converter has a variable input power from −40 to 20 dBm according to the received RF power and across a 50 kΩ resistive DC load. Investigations show that the number of stages leads to a high output power but, because of increased parasitic effects, the maximal efficiency remains almost constant, and is shifted to the higher input power region. Experimental results present a large divergence produced by PCB layer parasitic capacitance and the components’ tolerances. Results are compared to state of the art solutions and show an outstanding potential of power conversion efficiency.
